# Astrovirus Encephalitis in Boy with X-linked Agammaglobulinemia

**DOI:** 10.3201/eid1606.091536

**Published:** 2010-06

**Authors:** Phenix-Lan Quan, Thor A. Wagner, Thomas Briese, Troy R. Torgerson, Mady Hornig, Alla Tashmukhamedova, Cadhla Firth, Gustavo Palacios, Ada Baisre-De-Leon, Christopher D. Paddock, Stephen K. Hutchison, Michael Egholm, Sherif R. Zaki, James E. Goldman, Hans D. Ochs, W. Ian Lipkin

**Affiliations:** Columbia University, New York, New York, USA (P.-L. Quan, T. Briese, M. Hornig, A. Tashmukhamedova, G. Palacios, A. Baisre-De-Leon, J.E. Goldman, W.I. Lipkin); University of Washington, Seattle, Washington, USA (T.A. Wagner, T.R. Torgerson, H.D. Ochs); Seattle Children’s Hospital, Seattle (T.A. Wagner, T.R. Torgerson, H.D. Ochs); Pennsylvania State University, Pittsburgh, Pennsylvania, USA (C. Firth); Centers for Disease Control and Prevention, Atlanta, Georgia, USA (C.D. Paddock, S.R. Zaki); 454 Life Sciences, Branford, Connecticut, USA (S.K. Hutchison, M. Egholm)

**Keywords:** Astrovirus, encephalitis, immunodeficiency, unbiased high-throughput sequencing, astrocyte infection, neuronal death, viruses, research

## Abstract

Encephalitis is a major cause of death worldwide. Although >100 pathogens have been identified as causative agents, the pathogen is not determined for up to 75% of cases. This diagnostic failure impedes effective treatment and underscores the need for better tools and new approaches for detecting novel pathogens or determining new manifestations of known pathogens. Although astroviruses are commonly associated with gastroenteritis, they have not been associated with central nervous system disease. Using unbiased pyrosequencing, we detected an astrovirus as the causative agent for encephalitis in a 15-year-old boy with agammaglobulinemia; several laboratories had failed to identify the agent. Our findings expand the spectrum of causative agents associated with encephalitis and highlight unbiased molecular technology as a valuable tool for differential diagnosis of unexplained disease.

## Abstract 2

Unbiased pyrosequencing detected an astrovirus after conventional methods failed to identify the causative agent.

The economic cost of encephalitis is profound. Among the general population of western industrialized countries, the annual incidence of acute encephalitis is 7.3 cases per 100,000 persons (*1*). Although some persons recover from encephalitis without apparent sequelae, up to 71.0% experience lasting sequelae and up to 7.4% die (*1*,*2*). Khetsuriani et al. reported that each year in the United States alone, encephalitis is associated with ≈19,000 hospitalizations (average hospital stay 12 days), 1,400 deaths, and a cost of ≈$650 million for encephalitis-associated hospitalization (*1*).

Encephalitis is associated with a wide spectrum of infectious agents, including viruses, bacteria, fungi, and parasites (*3*). The most commonly implicated viruses are herpes simplex, varicella-zoster, Epstein-Barr, mumps, measles, and enteroviruses (*4*). Despite the use of various diagnostic methods (culture, molecular, immunohistochemical, or serologic), a causative agent is not identified for a high proportion of encephalitis cases (up to 75%) (*5*). This diagnostic failure may reflect the absence of a known agent or its molecular footprint at time of sampling, suboptimal specimen handling, lack of assay sensitivity, or presence of an unexpected or novel agent not considered in conventional assays. A better understanding of emerging and reemerging pathogens implicated in outbreaks of encephalitis (e.g., West Nile virus, Hendra virus, Nipah virus, Australian bat lyssavirus, and enterovirus 71) indicates an urgent need for novel tools for rapid differential diagnostic testing and surveillance (*6*,*7*).

The advent of unbiased molecular discovery technologies offers new opportunities to identify novel pathogens without the constraints imposed by assays selective for known or expected agents. We used unbiased high-throughput pyrosequencing to detect an astrovirus in a patient who died with unexplained encephalitis.

## Materials and Methods

### The Patient

In 2007, a 15-year-old boy with X-linked agammaglobulinemia (XLA) caused by a missense mutation (Thr35Pro) in the Bruton tyrosine kinase (*Btk*) gene was admitted to a psychiatric facility in Seattle, WA, USA, because of suicidal and homicidal ideation, headache, memory loss, and ataxia. He had progressive cognitive decline, was unable to walk or communicate within 4 weeks of admission, became comatose, and died 71 days after admission.

### Samples

Patient samples available for examination were fresh-frozen biopsy specimens of frontal cortex and postmortem tissues (brain stem, frontal lobe, kidney, liver, and spleen). Also available for immunohistochemical and neuropathologic examination were formalin-fixed postmortem samples of cerebral cortex, basal ganglia, and cerebellum from the patient and from other persons without encephalitis or brain inflammation.

### Unbiased High-Throughput Sequencing

RNA (0.5 μg) from the frontal cortex biopsy specimen was treated with DNase I (Ambion DNA-*free*; Austin, TX, USA) and reverse transcribed by using Superscript II (Invitrogen, Carlsbad, CA, USA) with random octamer primers linked to an arbitrary defined 17-mer primer sequence (Eurofins MWG Operon, Huntsville, AL, USA). cDNA was treated with RNase H before random PCR amplification with a 9:1 mixture of a 17-mer random sequence primer and an octamer-linked 17-mer random sequence primer (*8*). Products with >70 bp were purified by using MinElute (QIAGEN, Valencia, CA, USA) and ligated to linkers for sequencing on a GSL FLX Sequencer (454 Life Sciences, Branford, CT, USA). After primers were trimmed and highly repetitive sequences eliminated, reads were clustered and assembled into contiguous fragments for comparison by BLAST (*9*) with the GenBank database at the nucleotide and translated amino acid levels. We used custom software applications written in Perl (BioPerl 5.8.5) and programs available through the GreenePortal website (http://tako.cpmc.columbia.edu/Tools/).

### Rapid Amplification of cDNA Ends

Virus-specific primers for 5′ rapid amplification of cDNA ends were 5′-ACGCTCAAGCTCATGTCTGA-3′ for reverse transcription, 5′-GATGAGCGCTCTGTTTTCAA-3′ for the first PCR with UAP (Invitrogen), and 5′-TCAACCTCAACCCAATCGTT-3′ for the second PCR with AUAP (Invitrogen). Primers for 3′ rapid amplification of cDNA ends were 5′-CTCGCAAGGCATATGAGTGA-3′ and UAP (Invitrogen) for the first PCR and 5′-CTGGCTTGGTTGCAAAAGTT-3′ and AUAP (Invitrogen) for the second PCR. Final concentration of all primers was 0.2 mmol/L. PCR products were purified with QIAquick PCR Purification kits (QIAGEN) and directly dideoxy sequenced in both directions.

### Quantitative Real-Time PCR

Reactions were performed in an ABI 7300 cycler by using SYBR-Green Master Mix (Applied Biosystems, Foster City, CA, USA) with primers 5′-CCATGTGTCTGATGGTGCTG-3′ and 5′-TTGATCATATCAATCACCAAATCA-3′ in a volume of 25 μL. Cycling conditions were 50°C for 2 min and 95°C for 10 min, followed by 45 cycles at 95°C for 15 s and 60°C for 1 min.

### Cloning, Expression, and Purification of VP29

The VP29 variable region of the capsid gene (nt 5231–6562) was amplified by PCR with forward VP29-*Kpn*I primer 5′-CGGGGTACCTGCTAGGTAAATCAGCAAATACT-3′ and reverse VP29-XhoI primer 5′-CCGCTCGAGTTGATCATATCAATCACCAAATCA-3′. Histidine 6–tagged VP29 was expressed in *Escherichia coli* (Gateway vector pDEST 17; Invitrogen) and purified by using 1 mL Histrap column (GE Healthcare Life Sciences, Piscataway, NJ, USA) in denaturing binding buffer (20 mmol/L Tris-HCl [pH 8], 6 M guanidine, 0.5 M NaCl, 20 mmol/L imidazole), washed with 5 column volumes of denaturing binding buffer followed by 5 column volumes of denaturing wash buffer (20 mmol/L Tris-HCl [pH 8], 6 M urea, 0.5 M NaCl, 20 mmol/L imidazole), and eluted in denaturing elution buffer (20 mmol/L Tris-HCl [pH 8], 6 M urea, 0.5 M NaCl, 0.5 M imidazole). Peak fractions were analyzed by sodium dodecyl sulfate–polyacrylamide gel electrophoresis, pooled, and dialyzed against 20 mmol/L Tris-HCl (pH 8) and 3 M urea. Purification of VP29 was confirmed by Western blot with anti-histidine antibody (Genscript, Piscataway, NJ, USA) and mass spectroscopy of a trypsin-digested purified sample.

Rabbit antiserum against VP29 was generated by injecting rabbits with recombinant VP29 (3 injections of 0.5 mg each in Freund complete/incomplete adjuvant). Immunoglobulin (Ig) G was purified by using protein A-Sepharose (Lampire Biologic Laboratories, Pipersville, PA, USA).

### Immunohistochemistry and Immunofluorescence

Formalin-fixed, paraffin-embedded brain sections were heated at 56°C for 10 min, deparaffinized in a citrus clearing agent, and rehydrated through decreasing concentrations of ethanol. Endogenous peroxidase activity was blocked with 3% hydrogen peroxide for 15 min. Heat-induced antigen retrieval was performed in Trilogy antigen retrieval solution (Cell Marque, Rocklin, CA, USA) for 20 min at 95°C, after which the solution was cooled for 30 min. After blocking with Background Sniper solution (BS966H; Biocare Medical, Concord, CA, USA) for 10 min at room temperature, sections were incubated with primary antibodies overnight at 4°C. Slides were washed with wash buffer (Dako, Carpinteria, CA, USA) and incubated with appropriate secondary antibody for either immunohistochemical or immunofluorescence examination.

For immunohistochemical examination, Vectastain Elite ABC kits (PK-6101, AK5002; Vector Laboratories, Burlingame, CA, USA) were used to develop diaminobenzidine tetrahydrochloride chromogen. Tissue sections were incubated with either biotinylated goat antirabbit or biotinylated horse antimouse IgG (1:200, Vector Laboratories) for 1 h at 37°C, after which ABC reagents were added. Sections were counterstained with hematoxylin and dehydrated with 100% ethanol. Sections were affixed to slides by using Permount histologic mounting medium (Fisher, Fair Lawn, NJ, USA), and coverslips were placed.

For immunofluorescence assays, sections were incubated with secondary Cy3-conjugated goat antirabbit antibody or Cy2 goat antimouse antibody (1:200, Jackson ImmunoResearch Laboratories, West Grove, PA, USA) for 1 h at room temperature. Sections were mounted on slides by using ProLong Gold antifade reagent with DAPI (Invitrogen). Images were viewed on a Zeiss LSM 510 multiphoton confocal microscope and analyzed by using AIM Software (Carl Zeiss GmbH, Thornwood, NY, USA). Primary antibodies used were mouse antiglial fibrillary acidic protein cocktail (1:100, BD Bioscience Pharmagen, San Jose, CA, USA), mouse anti-CD3 (1:350, Dako), and mouse anti-CD68 (1:50, Dako).

### Phylogenetic Analysis

Representative capsid gene (open reading frame 2) sequences were downloaded from GenBank, and aligned with the capsid gene sequence of the novel astrovirus by using Se-Al version 2.0a11 (http://tree.bio.ed.ac.uk/software/seal/). A Bayesian phylogenetic tree based on the full-length amino acid alignment of the capsid protein was generated by using MrBayes version 3 (*10*) and the WAG amino acid transition model. Two independent runs were allowed to converge over 10 million generations, burn-in setting was 10%, and every 1,000 generations were sampled. The robustness of the resultant phylogenetic analysis was assessed by using Bayesian posterior probability values.

## Results

During the patient’s hospitalization, Gram stain, bacterial cultures, PCR, and cryptococcal antigen tests of 2 cerebrospinal fluid samples performed on days 6 and 12 after hospitalization failed to detect any agents that might be implicated in disease (Table 1; Table 2 Table 3; Table 4). Examination of a frontal cortex biopsy found diffuse astrogliosis and microgliosis of gray and white matter, perivascular and parenchymal CD3+ T-cell infiltrates, neuronal loss, and axonal swelling. No Negri bodies, Cowdry inclusions, or evidence of prion disease were found. No infectious agents were detected by electron microscopy or immunohistochemistry when a panel of antiserum for detection of herpes simplex virus, polyomavirus, and adenovirus was used. Bacterial and fungal cultures, broad-based 16S rRNA PCR, viral culture, and PCR for multiple viruses were negative (Table 2; Table 3; Table 4).

**Table 1:** Results of testing of cerebrospinal fluid from 15-year-old boy with X-linked agammaglobulinemia and astrovirus encephalitis*

Hospitalization day	Nucleated cells, cells/mm3 (ref <5)	Erythrocytes, cells/mm3	Neutrophils, %	Protein, mg/dL (ref <40)	Glucose, mg/dL (ref 45%–60% of glucose in serum)	Immunoglobulin G, mg/dL (ref 0.8–7.7)
6	4	2	2	76	50	ND
12	6	107	1	48	57	3.7

*Ref, reference value; ND, not done.

**Table 2:** Staining and culture results for 15-year-old boy with X-linked agammaglobulinemia and astrovirus encephalitis*

Sample	AFB stain	Gram stain	Cryptococcal antigen	Bacterial culture†	Fungal culture	Viral culture
Cerebrospinal fluid	ND	Neg	Neg	Neg	ND	ND
Brain biopsy	Neg	ND	ND	Neg	Neg	Neg

*AFB, acid-fast bacteria; ND, not done; neg, negative. †Includes Mycobacteria spp.

**Table 3:** PCR results for bacteria, fungi, parasites, and DNA viruses in 15-year-old boy with X-linked agammaglobulinemia and astrovirus encephalitis*

Sample	Bacteria (16S)	Fungi	Toxoplasma gondii	Adenovirus	BK virus	CMV	Epstein-Barr virus	JC virus	Parvovirus B19
Cerebrospinal fluid	ND	ND	ND	ND	Neg	Neg	Neg	Neg	ND
Brain	Neg	Neg	Neg	Neg	Neg	Neg	Neg	Neg	Neg

*CMV, cytomegalovirus; ND, not done; neg, negative.

**Table 4:** PCR results for RNA viruses in 15-year-old boy with X-linked agammaglobulinemia and astrovirus encephalitis*

Sample	Coronavirus	Enterovirus	HMPV	HPIV 1 to 4	HSV 1 or 2	Influenza virus†	Rhinovirus	RSV	VZV	WNV
Cerebrospinal fluid	ND	Neg	Neg	ND	Neg	ND	ND	ND	Neg	ND
Brain	Neg	Neg	Neg	Neg	Neg	Neg	Neg	Neg	Neg	Neg

*HMPV, human metapneumovirus; HPIV, human parainfluenza virus; HSV, herpes simplex virus; RSV, respiratory syncytial virus; VZV, varicella zoster virus; WNV, West Nile virus; ND, not done; neg, negative. †Type A or B.

**Figure 1:**
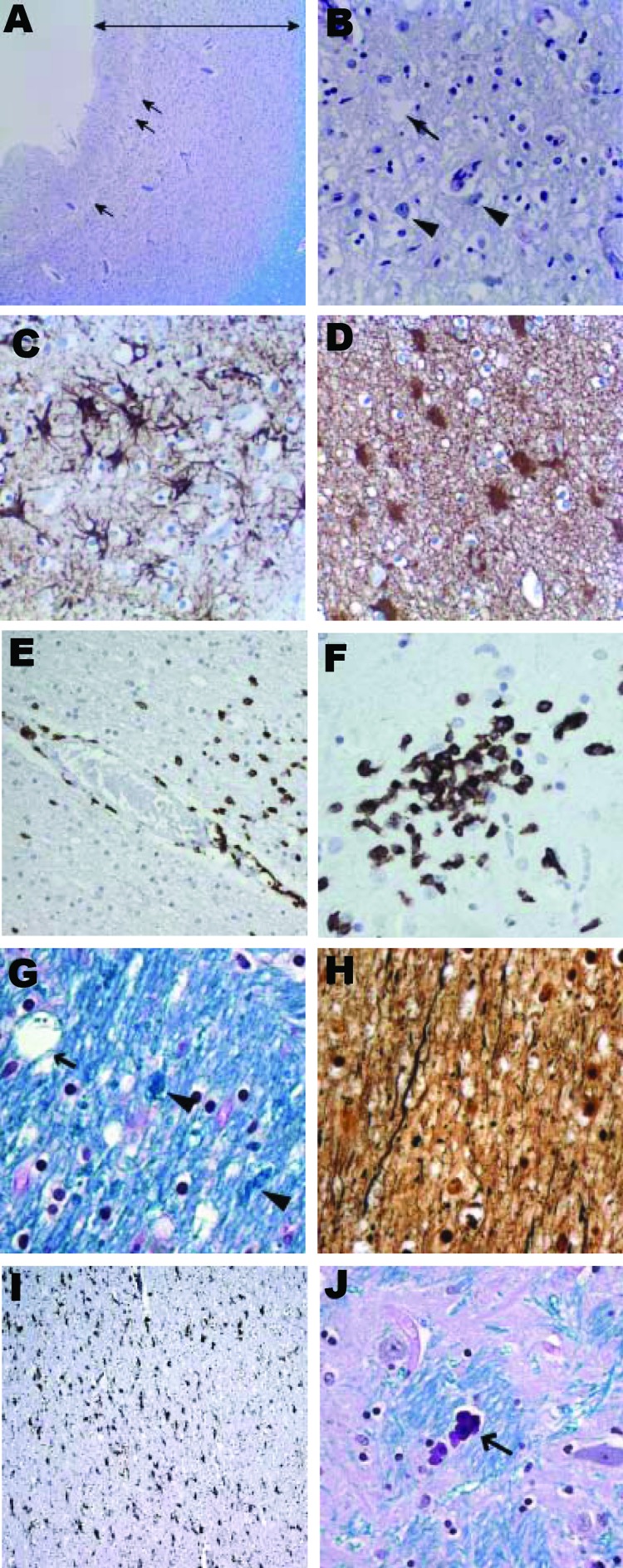
Histologic findings from brain of 15-year-old boy with X-linked agammaglobulinemia and astrovirus encephalitis. A) Frontal cortex with cortical thinning (double-headed arrow) and vacuolation (arrows) (Luxol fast blue stain with periodic acid–Schiff method [LFB/PAS], original magnification ×10). B) Frontal cortex with vacuolation (arrow) and rare residual neurons (arrowheads) (LFB/PAS, original magnification ×50). C) Marked astrogliosis in the frontal cortex (glial fibrillary acidic protein [GFAP] immunostain, original magnification ×100). D) White matter with marked astrogliosis (GFAP immunostain, original magnification ×100). E) Penetrating artery with abundant CD3+ T-cells in the perivascular space and adjacent brain parenchyma (CD3^+^ immunostain, original magnification ×25). F) CD3^+^ T-cells as part of microglial nodules (CD3^+^ immunostain, original magnification ×100). G) White matter in the internal capsule showing myelin clumps (arrowheads) and vacuolation (arrow) (LFB/PAS, original magnification ×100). H) Loss of axons in the internal capsule (Bielschowsky nerve fiber silver stain, original magnification ×100). I) Internal capsule with marked microgliosis (CD68^+^ immunostain, original magnification ×40). J) Microcalcifications (arrow) in the globus pallidus (LFB/PAS, original magnification ×100). All paraffin sections were counterstained with hematoxylin.

Histologic examination of postmortem brain specimens showed diffuse neuronal loss, cortical thinning, and vacuolation of the gray matter of the cortices and deep nuclei (Figure 1, panels A, B) accompanied by astrogliosis in cortex and subcortical white matter (Figure 1, panels C, D). CD3^+^ T-lymphocytes were identified in the perivascular spaces, infiltrating the parenchyma (Figure 1, panel E), and in microglial nodules (Figure 1, panel F). No CD20^+^ B cells were noted. The white matter showed vacuolation and myelin clumps consistent with myelin degeneration (Figure 1, panel G), axonal loss (Figure 1, panel H), and microgliosis (Figure 1, panel I). Microcalcifications were seen in the globus pallidus (Figure 1, panel J).

**Figure 2:**
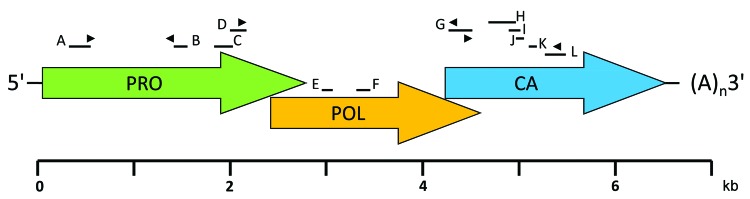
Schematic genome organization of human astrovirus Puget Sound (HAstV-PS). Arrows represent the 3 open reading frames of the 6,584-nt single-strand, positive-sense genome. Bars above the schematic indicate the 12 contiguous fragments (contigs A–L) generated through unbiased high-throughput sequencing. PCR primers for amplification across sequence gaps were designed based on the unbiased high-throughput sequencing data, and the draft genome was resequenced by overlapping PCR products that covered the entire genome except for terminal sequences. Genomic termini were characterized with 5′ and 3′ rapid amplification of cDNA ends kits (Invitrogen, Carlsbad, CA, USA). Arrowheads indicate primer locations. PRO, protease; POL, polymerase; CA, capsid; (A)_n_, poly-A tail.

**Figure 3:**
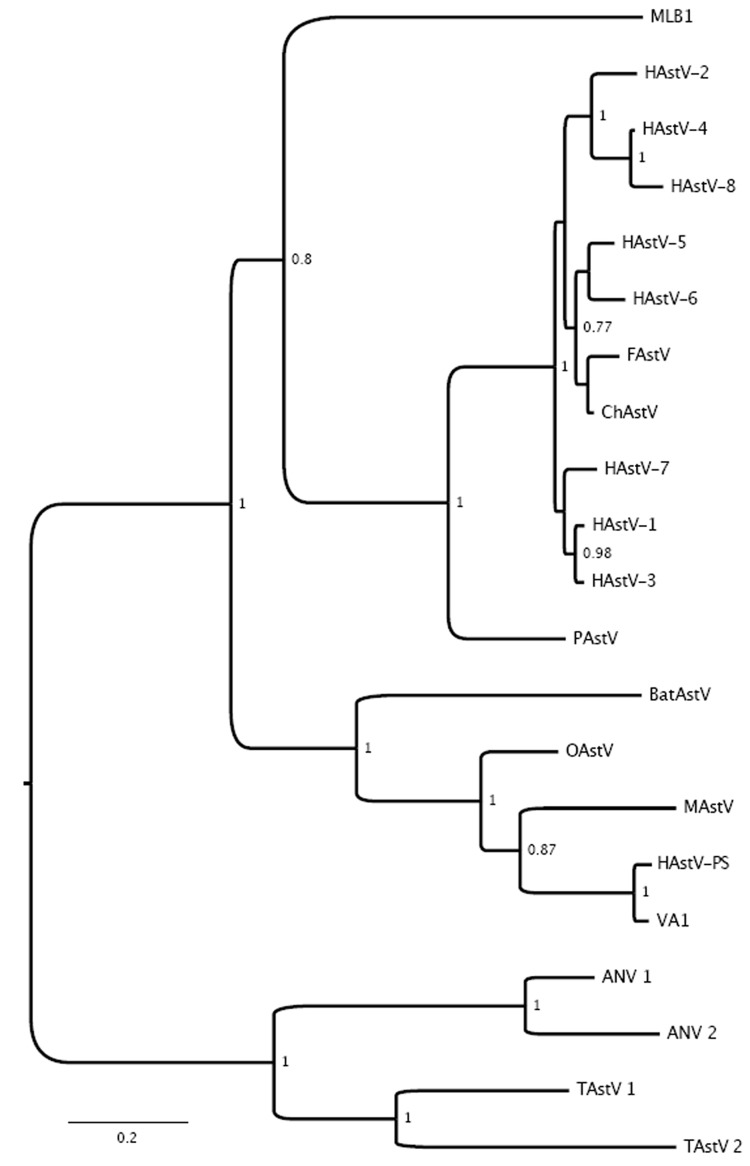
Phylogenetic analysis of full-length capsid protein sequences showing the relationship between human astrovirus Puget Sound (HAstV-PS) identified in brain of 15-year-old boy with X-linked agammaglobulinemia and encephalitis and other astroviruses. GenBank accession numbers in parentheses: MLB1 (FJ22245), VA1 (FJ973620), HAstV-1 (AB000295), HAstV-2 (L06802), HAstV-3 (DQ630763), HAstV-4 (AB025803), HAstV-5 (U15136), HAstV-6 (Z46658), HAstV-7 (Y08632), HAstV-8 (Z66541), MAstV (AY179509), OAstV (NC_002469), BatAstV (FJ571074), FAstV (AF056197), ChAstV (EU650331), PAstV (Y15938), ANV 1 (AB033998), ANV 2 (AB046864), TAstV 1 (Y15936), and TAstV 2 (AY769615). Bayesian posterior probability values >75% are shown at respective nodes. FAstV, feline astrovirus; ChAstV, cheetah astrovirus; PAstV, porcine astrovirus; OAstV, ovine astrovirus; MAstV, mink astrovirus; ANV, avian nephritis virus; TAstV, turkey astrovirus. Scale bar indicates number of amino acid substitutions per site.

Because the histologic findings were consistent with virus infection and because molecular, serologic, and morphologic methods failed to identify an infectious agent, we pursued unbiased pyrosequencing of RNA from the frontal cortex biopsy specimen (*11*,*12*). From 2 cDNA libraries that mapped either to human (host) sequences or were uninformative in GenBank searches at the nucleotide level (BLASTn), we obtained an average of 102,000 sequence fragments with a mean length of 180 nt. However, analysis at the deduced amino acid level (BLASTx) identified 12 sequences homologous to astroviruses (Figure 2; Table 5). The complete 6,584-nt genomic sequence of the virus (GenBank accession no. GQ89199) was determined in a 2-step procedure in which gaps between sequence fragments identified through pyrosequencing were filled by PCR amplification and sequencing, and the genomic termini were cloned and sequenced by rapid amplification of cDNA ends (Figure 2; Table 6). Phylogenetic analyses placed the virus, tentatively named human astrovirus Puget Sound (HAstV-PS), apart from known human astroviruses. Analysis of the full-length capsid protein sequence showed HAstV-PS in a clade together with ovine, mink, and bat astroviruses (Figure 3); analyses of polymerase or protease protein sequences gave comparable results (data not shown).

**Table 5:** Genomic location of HAstV-PS contigs identified by 454 pyrosequencing and their relationship to known astroviruses*

Contiguous fragment	Genome position in HAstV-PS	Highest amino acid identity, %	GenBank accession no.†	Astrovirus species
A	238–504	52	NP_795334	Mink
B	1765–1839	64	NP_795334	Mink
C	2012–2184	67	NP_795334	Mink
D	2308–2466	42	NP_059945	Ovine
E	2763–2945	48	AAO32082	Mink
F	3360–3728	65	NP_059945	Ovine
G	4558–4679	65	NP_795336	Mink
H	4558–5140	70	NP_059946	Ovine
I	4708–4944	72	NP_795336	Mink
J	4801–5110	72	NP_059946	Ovine
K	4819–4929	76	NP_059946	Ovine
L	5104–5331	70	NP_059946	Ovine

* HAstV-PS, human astrovirus Puget Sound. †Accession number for BLASTX (www.ncbi.nlm.nih.gov/blast/Blast.cgi) match with highest amino acid identity (GenBank database December 2007).

**Table 6:** Primers used in cloning and sequencing the human astrovirus Puget Sound genome

Sequence, 5′ → 3′	Position
TCATGGAGCGCTCATACAAG GTGTAAGCGAAGCCAAAAGC	38–57 815–796
GATTGGGTTGAGGTTGATGC* TCCAGTGGTGGCTTGATGTA	340–359 1797–1778
GAAGTGGGATGGTGGAGTTG TCCAGAAATCCGATTCAACC	666–685 1314–1295
TGGAGCAGTTGTTGGTGAAA TTCTCAAGGTCTATCTCCCTTTGT	1215–1234 1877–1854
TACATCAAGCCACCACTGGA* TCCGCCTCTCTAAGCACTTC	1778–1797 2159–2140
TACATCAAGCCACCACTGGA TTGGATTGACTCCCTCAAGC	1778–1797 2533–2514
ATCACCACAAGAGGCGTAGG* CCAGTAACTGCTGATGGACCAACAA	2039–2058 4649–4625
CGTGATTTGCAGGAATACCA CCCATACGTGTCAGGGTTCT	2446–2465 3343–3324
TCTGGAGAGAGGCCTGATGT TCTCTCTTGACCCACATCCC	3159–3178 3865–3846
CCTCTGGGCAAATATCCACA TAGGGATATGCGGAAAACAGA	3625–3644 4671–4651
GTTTGTGGCGCTTGAAAAGT TTGCGTGGAAGAAAGTGTTG	4583–4602 5365–5345
TTGTTGGTCCATCAGCAGTTACTGG* AACTTTTGCAACCAAGCCAG	4 625–4649 5175–5156
CTGGCTTGGTTGCAAAAGTT GAGTATTGTGCCCGCAAAGT	5156–5175 6041–6022
CGGCTACACCAGCCTACATT CAGCACCATCAGACACATGG	5833–5852 6442–6423

*Primer pairs designed for amplification between ultra–high-throughput sequencing contiguous fragments (Figure 2); primer pairs without asterisk were applied for resequencing of the draft genome.

Real-time PCR indicated that viral load was higher in the biopsy specimen (1.53 × 10^7^ RNA molecules per reaction) than in postmortem specimens (cerebellum [5.39 × 10^2^], frontal lobe [1.14 × 10^2^], and brain stem [1.92 × 10^4^]). No viral RNA was found in postmortem kidney, liver, or spleen specimens.

**Figure 4:**
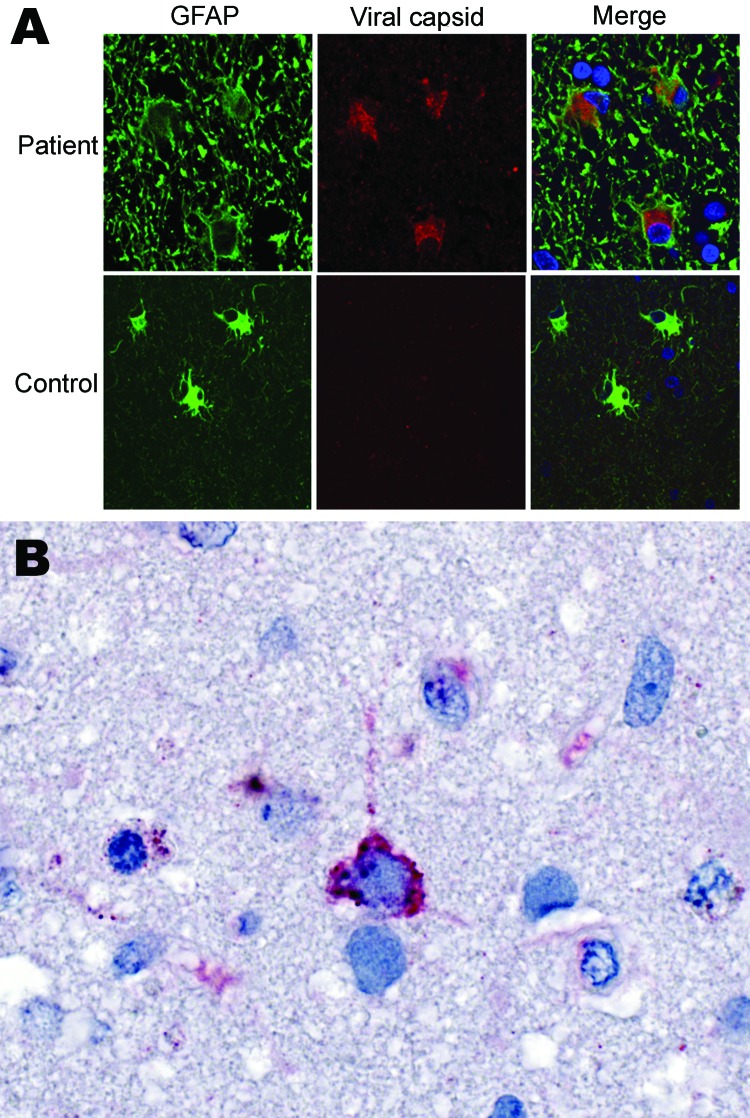
Immunofluorescence and immunohistochemical analyses with human astrovirus Puget Sound capsid antibodies. A) Indirect double immunofluorescence–stained, formalin-fixed, paraffin-embedded tissue sections from 15-year-old boy with X-linked agammaglobulinemia and astrovirus encephalitis and a control with astrogliosis not caused by astrovirus infection. The sections were stained for the astrocyte marker glial fibrillary acidic protein (GFAP, green) and for viral capsid protein (rabbit serum 1:1,000, red). Viral capsid protein is present in hypertropic astrocytes throughout the subcortical white matter and cortex; astrocytes have swollen cell bodies with intense GFAP immunostaining. Blue signal (DAPI) indicates nuclear counterstaining. Original magnification ×100. B) Immunohistochemical localization of viral antigen in a frontal cortex biopsy specimen. Immunoalkaline phosphatase stain with viral capsid antibodies **(**rabbit serum 1:1,000) and naphthol-fast red with hematoxylin counterstain. Original magnification ×158.

Commercial antibodies to human astroviruses did not stain infected brain (data not shown). Therefore, polyclonal antibodies were generated by injecting rabbits with recombinant capsid protein of HAstV-PS. Indirect double-immunofluorescence staining of postmortem tissue sections demonstrated capsid protein in hypertropic astrocytes throughout the subcortical white matter and cortex (Figure 4, panel A). Astrocytes had swollen cell bodies and showed intense immunostaining of the glial fibrillary acidic protein. Indirect immunohistochemical staining of a frontal cortex brain biopsy specimen demonstrated intracytoplasmic capsid protein in only 1 cell; morphologic appearance was consistent with that of a neuron (Figure 4, panel B). No evidence of infection was found in oligodendrocytes or macrophages.

## Discussion

The astrovirus identified in the central nervous system (CNS) of an immunocompromised patient with XLA and encephalitis was discovered through unbiased high-throughput pyrosequencing after conventional methods failed to identify an infectious agent. The astrovirus infection was confirmed by specific PCR and antigen detection. To prove causation according to the Koch postulates, the infectious agent must be propagated and must reproduce disease in a previously unexposed host (*13*); to prove causation according to Rivers, a specific humoral immune response to infection must be found (*14*). Because we have not been able to grow the astrovirus in culture and because persons with XLA do not generate specific antibodies, these criteria have not been met. Nonetheless, the neuropathologic findings consistent with viral encephalitis in conjunction with the high viral load found in the CNS suggest a causative association between HAstV-PS and disease. The most prominent histologic lesions in the brain consisted of severe neuronal degeneration, hypertrophic astrocytes, and infiltration by T-lymphocytes and macrophages. Although we cannot exclude prominent neuronal infection earlier in the course of infection, immunohistochemical and immunofluorescence studies localized astroviral protein mainly in astrocytes.

Astrocytes are essential to neuronal function and viability (*15*); they are critical for maintenance of the blood–brain barrier; they provide axon guidance during development and structural support to neural elements; and they are involved in CNS homeostasis as regulators of extracellular glutamate, ionic environment, and pH (*16*). Astrocyte dysfunction is implicated in the pathogenesis of acute and chronic CNS disorders (*16*,*17*). Recent data indicate that infected astrocytes play a role in the pathogenesis of HIV-associated dementia, a neurodegenerative disorder (*18*). The essential features of this disorder are cognitive and motor impairment, speech problems, and behavioral changes. Similar to findings for the patient described here, HIV-associated dementia is characterized by infiltration of macrophages into the CNS, gliosis, pallor of myelin sheaths, and loss of neurons (*19*).

Astroviruses have not previously been associated with CNS disease. They are nonenveloped, single-stranded RNA viruses, are typically transmitted by the fecal–oral route, and cause mild gastrointestinal disease. Antibodies to human astrovirus-1 have been found in >90% of the human population (*20*). Immunocompromised persons are prone to astrovirus infections of the gastrointestinal tract (*21*). Postmortem analysis of the gastrointestinal tract of the patient described in this article found no evidence of astrovirus infection (data not shown); however, we cannot exclude the possibility of a gastrointestinal infection that cleared before the patient died.

The fact that the patient had XLA may explain dissemination of the astrovirus to the CNS. XLA is a primary immunodeficiency disorder caused by mutations in the *Btk* gene, which results in absence of B lymphocytes and serum immunoglobulins (*22*). Several recent reports demonstrate that Btk is required for Toll-like receptor 8–mediated production of interleukin-6 and production of tumor necrosis factor-α by peripheral blood mononuclear cell–derived dendritic cells (*23*,*24*). Hence, Btk deficiency may impair innate immune responses after a person is infected with single-stranded RNA viruses known to cause fatal CNS infection in those with XLA (*25*,*26*), such as enteroviruses, and now, potentially, astroviruses.

The source of infection for the patient described here remains unknown. Exposure to mink was a potential source, suggested by the phylogenetic relationship of HAstV-PS to mink astroviruses and the proximity of the patient’s residence to a mink farm. Another possible source was the patient’s monthly treatment with intravenous immunoglobulin. Several reports describe progressive neurodegeneration of unknown cause in immunosuppressed patients who received long-term intravenous immunoglobulin therapy (*26*,*27*). Some of these patients had neuropathologic findings similar to those reported here (*26*,*27*). Intravenous immunoglobulin preparations from 5 companies (Vivaglobin [CSL Behring GmbH, Marburg, Germany], Carimune [CSL Behring GmbH], Gammagard [Baxter, Westlake Village, CA, USA], Gamimune N [Bayer Healthcare Pharmaceuticals, West Haven, CT, USA], Flebogamma [Instituto Grifols, S.A. Barcelona, Spain]) were negative for the newly identified astrovirus, according to ELISA and PCR (data not shown).

The recent finding of an astrovirus closely related to HAstV-PS in fecal samples from children from Virginia with acute gastroenteritis (*28*) suggests that these novel viruses are circulating widely in humans across the United States. They should be considered in the differential diagnosis of encephalitis, particularly for immunosuppressed patients.

Despite extensive microbiologic investigation, the causes of up to 75% of encephalitis cases remain elusive. These undiagnosed cases pose a challenge for clinical medicine and public health and underscore the need to invest in developing new methods for investigating these debilitating, frequently fatal, disorders. To address the challenge of unexplained encephalitis, public health practitioners and diagnosticians need a more comprehensive armamentarium and methods that would enable them to discover new and unexpected pathogens associated with encephalitis. Our findings emphasize the value of unbiased pyrosequencing as a powerful tool for diagnosing the cause of encephalitis and the need to consider astroviruses as CNS pathogens, particularly in immunosuppressed persons. Early recognition of the causative agent of unexplained encephalitis cases will enable specific interventions that reduce illness and death and facilitate the recognition of outbreaks that threaten public health.
